# The neural underpinnings of repeated skill transfer in human cultural evolution

**DOI:** 10.3389/fpsyg.2025.1545120

**Published:** 2025-05-07

**Authors:** Heidi Øhrn, Emilie Pettersen Sjursen, Karsten Specht, Kenneth Hugdahl, Larissa Mendoza Straffon, Andrea Bender

**Affiliations:** ^1^Department of Psychosocial Science, Faculty of Psychology, University of Bergen, Bergen, Norway; ^2^SFF Centre for Early Sapiens Behaviour (SapienCE), University of Bergen, Bergen, Norway; ^3^Department of Biological and Medical Psychology, Faculty of Psychology, University of Bergen, Bergen, Norway; ^4^Mohn Medical Imaging and Visualization Centre (MMIV), Haukeland University Hospital, Bergen, Norway; ^5^Division of Psychiatry, Haukeland University Hospital, Bergen, Norway

**Keywords:** cumulative cultural evolution, social learning, knot-tying, cognitive evolution, observational learning, imitation, fMRI, transmission chain

## Abstract

Cumulative cultural evolution (CCE) is a fundamental aspect of human cognition, enabling the refinement and transmission of complex skills across generations. This study explores the cognitive abilities supporting CCE through a transmission chain design using a knot-tying task combined with brain imaging to examine how skills are acquired over successive learning and transmission stages. We obtained data from two chains of multiple generations of participants. Our results revealed generational modifications in knot-tying techniques accompanied by increased prefrontal cortex activation in later generations of learners, possibly suggesting that loss of information due to imperfect copying fidelity increases cognitive demands for working memory. Our study further shows the potential of brain imaging as a viable technique for investigating CCE. By applying functional MRI to track neural activity during the acquisition of knot-tying skills, we provide a novel approach for understanding the cognitive mechanisms that underlie cultural knowledge transfer. Further research integrating neuroimaging with behavioral studies could help clarify how cognitive and neural processes contribute to the accumulation and refinement of cultural knowledge over time.

## 1 Introduction

The material artifacts left behind by prehistoric humans open a window into the mental and cultural lives of those that came before us (Currie and Killin, 2019). The complexity and richness of human culture is believed to be, at least in part, the result of cumulative cultural evolution (CCE). CCE is described as a process of repeated cultural transmission, during which small changes in behavior can spread between the individuals in a population, and over time accumulate in a “ratchet like” manner (Tennie et al., 2009). When these modifications have positive consequences for the population they usually stay in place until a newer one is introduced (Tomasello et al., 1993). Cultural transmission is, in turn, powered by mechanisms of social learning, such as imitation and teaching, which contribute to copying fidelity and the accurate reproduction of behaviors (Caldwell et al., 2018). The accumulation of skills and knowledge generated by CCE allows younger generations to benefit from the innovations made by the generations preceding them, leading to behaviors and artifacts that no single individual would have been able to invent on their own (Tennie et al., 2009). CCE is therefore believed to be a major driver for human cognitive evolution (Boyd and Richerson, 1996; Tomasello, 1999).

Although social learning is foundational to CCE in general, the form of social learning deployed in the successful transmission of behavior will vary according to several factors, including task complexity, the number of accessible models, and the amount of time available for solving a task. For instance, in simple tasks, emulation (i.e., focusing on the end product or effect rather than on copying the process) may be sufficient to achieve the successful transmission of culture (Caldwell and Millen, 2009; Zwirner and Thornton, 2015). However, with increasing task complexity, forms of social learning that support high-fidelity reproduction, such as imitation and teaching, become more beneficial and ultimately necessary for CCE to happen (Dean et al., 2012; Wasielewski, 2014; Zwirner and Thornton, 2015). Such differential use of social learning may be because complex artifacts and behaviors tend to be ‘opaque’ (information about how they work or are produced is not easily extracted by direct observation), whereas simple ones can be more easily ‘reverse-engineered’, allowing individuals to reproduce them through different techniques. Therefore, we would expect the development of ever more complex cultural artifacts to increase selective pressures for social learning strategies over time (Wasielewski, 2014). One would also expect populations to improve ways to support the social transmission of knowledge and complex skills, such as increasing both access to experts as well as opportunities to learn from multiple models (Muthukrishna et al., 2014).

The prime means for investigating the cognitive mechanisms underlying CCE in the lab are transmission chain studies, in which knowledge or skills are repeatedly transferred from one individual to the next (Caldwell and Millen, 2008a). In a transmission chain design, the first participant of a chain watches a trained model performing a behavior, for example building a Lego house, and is instructed to recreate it. This participant will then become the model for the next in line, and so on, so that every participant in the chain follows the one before and models for the one after, each of them representing a new ‘generation’. Despite their limitations, these types of experiments can illuminate whether and in what ways cumulative culture occurs under different conditions (e.g., with/without verbal instruction, long/short observation times; modeling from naive or expert individuals, etc.) and involving different objects or behaviors (e.g., transparent or opaque, simple or complex, novel or familiar, etc.) (Muthukrishna et al., 2014; Papa et al., 2021; Wasielewski, 2014; Zwirner and Thornton, 2015).

Another approach to investigating the production, transmission and evolution of human culture is offered by the emerging field of neuroarchaeology. This is a multidisciplinary field that uses neuroscientific methods to help answer archeological questions (Salagnon et al., 2020). By having modern humans recreate artifacts found in the archeological record, we can in principle identify which skills and neural substrates are required for their perception and production, and infer when and how some human cognitive abilities evolved (Salagnon et al., 2020; Stout et al., 2015). The production of stone tools through flint knapping has been widely investigated in neuroarchaeology because stone tools are well preserved and plentiful in the archeological record, offering a material basis to trace human cognitive and cultural evolution (Soffer and Adovasio, 2010; Hurcombe, 2014). Flint knapping refers to the action of hitting one stone with another stone or hard material to gradually remove flakes and shape the stone into a tool. Looking at stone tools from different time periods reveals a trend toward increased processing of the raw material leading to more complex and symmetrical end products (Stout et al., 2008).

Flint knapping is a process that requires a combination of perceptual motor coordination and conceptual understanding, and it takes years of training to become a proficient knapper (Stout et al., 2008). The amount of time and effort needed to learn flint knapping suggests that the acquisition of this skill is dependent on social learning and support (Fragaszy et al., 2013). This was demonstrated by Morgan et al. (2015) when testing the effectiveness of different forms of social learning in transferring simple flint knapping skills. Their study indicated that transmission chains with some form of teaching, and especially teaching that included speech, showed improved skill acquisition relative to chains that relied on reverse engineering. Stout and Hecht (2017) have highlighted how adaptations for visuomotor integration laid the evolutionary foundation for the high-fidelity social learning involved in CCE. They suggest that adaptations and connectivity between the ventral premotor cortex, middle temporal visual areas, and parietal areas made humans capable of integrating increasingly detailed action information and complex goals while observing and imitating others (Stout and Hecht, 2017). The intraparietal sulcus (IPS) and the supramarginal gyrus (SMG) are some of the functional regions supporting such integrations, and are recruited during stone tool-making activities (Stout and Chaminade, 2007; Stout et al., 2011).

The increase in complexity observed in stone tool technologies over evolutionary time is theorized to correlate with the evolution of higher-order cognitive functions (e.g., sustained attention, planning, working memory) that allow the knapper to follow a planned sequence of intentions and actions (Stout et al., 2008). The evolutionary link between the production of increasingly complex stone tools and higher-order cognitive functions is further supported by brain imaging studies showing activation in the left dorsolateral prefrontal cortex (DLPFC) during the production of complex stone tools such as handaxes, but not simpler stone tools such as choppers and cleavers (Stout and Chaminade, 2007; Stout et al., 2008; Putt et al., 2017; Putt et al., 2019). The DLPFC is associated with working memory and other executive functions related to planning and conducting a sequence of goal-directed actions (Fuster, 2001). The differences in neural activation indicate an increased demand for effective visuomotor coordination and hierarchical action observation in more advanced stone toolmaking (Stout and Chaminade, 2007; Stout et al., 2008; Putt et al., 2017; Putt et al., 2019).

Similar to stone tool production, knot-tying is likely to have been an important subsistence skill for prehistoric humans. Although direct evidence of knots is a lot less prevalent in the archeological record than stone tools, we have indirect evidence that knots have been in use for at least 90,000 years (Kuhn and Clark, 2014; Kaaronen et al., 2024). The biggest indication comes from the invention of hafting to make composite tools. Fastening sharp-edged knapped stones or points to shafts of wood or bone by means of a knotted string allowed for the production of new tools such as spears, knives, and axes. Hafting involved a radical departure from the reductive process of stone knapping toward an additive and hierarchical process that pulls together separate components to form a new whole (Barham, 2013). Other possible uses of rope and knots include the making of snares and nets to gather food, carrying implements such as baskets, and the creation of fabrics that could be worn, or strung up to give shelter from the elements (Hardy et al., 2020).

The emergence of symbolic artifacts marks another milestone in human cognitive and cultural evolution. One of the earliest forms of symbolic material culture is found in the form of shell beads that were strung and knotted together to be used as body ornamentation (Vanhaeren et al., 2013; Bar-Yosef Mayer et al., 2020). The best-known example of such artifacts comes from Blombos Cave in South Africa, dating to c. 75,000 years ago (Henshilwood et al., 2004). Similar shells, purportedly used as beads, have been found in several sites along the present coast of South Africa, North Africa and the Levant, with the earliest ones going as far back as 140–120,000 years ago (Vanhaeren et al., 2006; Sehasseh et al., 2021). Even considering the dating uncertainty, the record indicates that by 100,000 years ago humans had started collecting shells and fastening them, likely on knotted strings, to be used as decoration (Vanhaeren et al., 2013).

**Figure 1:**
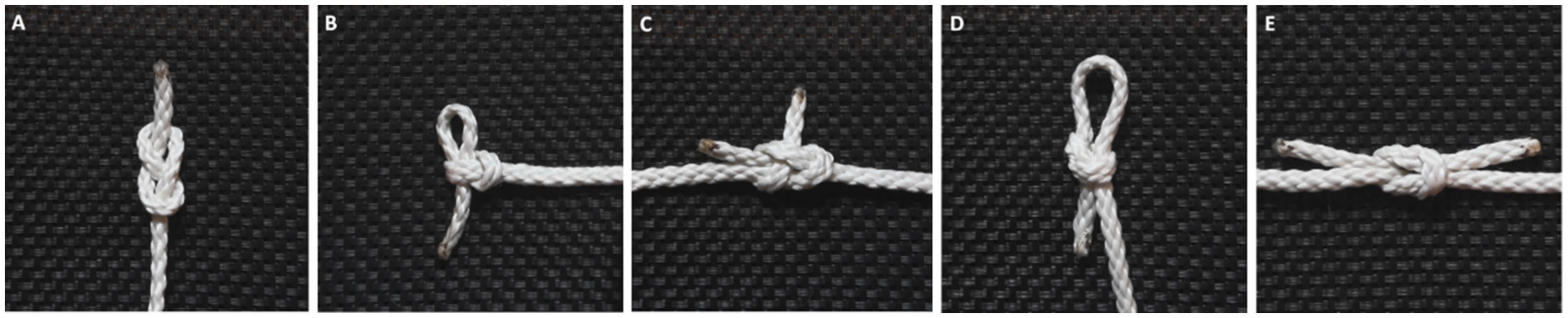
The five knots participants were tasked with learning: the figure eight knot **(A)**, noose knot **(B)**, sheet bend **(C)**, slip knot **(D)**, and square knot **(E)**.

Both knot-tying and the production of string are cognitively sophisticated activities that involve a complex chain of operations, requiring capacities for planning and working memory, and possibly incipient mathematical understanding (Hardy et al., 2020; Gerdes, 2010; Hurcombe, 2014). In a recent cross-cultural review, Kaaronen et al. (2024) found that several types of knots appear recurrently in various cultures and geographical locations. They further noticed that the use of certain kinds of knots cluster in geographical regions, leading to the assumption that knowledge about knots is culturally transmitted by social learning. On a global scale, the most common knots were the sheet bend (Figure 1C), the square knot (reef knot) (Figure 1E), the overhand knot, and the cow hitch. Interestingly, the most prevalent knot across cultures was not the simplest one (the overhand knot) but the more complex sheet bend knot, perhaps due to its efficiency and strength.

As mentioned above, transmission chain studies have become standard for studying different aspects of CCE and have proven to be informative about the factors that affect cultural evolutionary processes, such as task opacity, access to information, and learning mechanisms (Caldwell, 2020). However, it is difficult to pinpoint why/how these factors lead to different outcomes. Neuroarcheological approaches, for their part, have been used to reveal the cognitive demands of learning and transmission in the technological domain (Stout, 2021). Thus, applying brain imaging during the process of the cultural transmission of information and knowledge might help us get a better understanding of the mechanisms at play in CCE.

## 2 The present study

In this study, we combined functional magnetic resonance imaging (fMRI) with a transmission chain paradigm, with the aim to deepen our understanding of the underlying mechanisms of CCE. We chose the acquisition and transmission of knot-tying skills as our experimental task. Unlike stone tool knapping experiments which are costly, risky, and time-consuming (Stout et al., 2008), knot-tying can be learned relatively quickly, involves low-cost materials, is amenable to a lab environment, and can be performed in an fMRI machine, allowing us to record neural activity throughout the entire skill acquisition process. At the same time, knots are assumed to be somewhat opaque, so the acquisition of knot-tying skill should rely on high-fidelity social learning (Kaaronen et al., 2024). All of this made knots a desirable stimulus, while being no less cognitively, culturally, or evolutionarily relevant than stone tools as a technology.

The experimental task consisted of learning how to tie five knots. While in the MRI scanner, participants were shown step-by-step videos of how to tie each knot and practiced physically tying the knot themselves. After the imaging session, the participants demonstrated the tying of each knot. The demonstration was filmed, and the video was used as instruction for the following participant, thereby creating a transmission chain of information.

Behavioral transmission chain studies in which participants replicate a target artifact have shown that repeated transmission leads to changes in said artifact, either through loss of information (Morgan et al., 2015), or accumulation of helpful changes (Caldwell and Millen, 2008b). On that basis, the main research question of this study concerned whether the behavioral changes of learning, experience, and the repeated transfer of information would be reflected in the neural activation patterns of our participants, and if so, whether these might aid identifying neural regions involved in the transmission of cultural information. To our knowledge, no previous studies have looked at neural activity during the process of the repeated transmission of skills. We chose fMRI because this method provides full brain images. Although it did not allow for direct contact between participants, each individual was still embedded in a chain via video demonstrations from a previous participant, ensuring some degree of social learning.

We formulated three main hypotheses based on the literature on transmission chain studies and neural activation patterns during stone toolmaking and knot-tying. (i) We expected to find a general effect of acquiring the knot-tying skill when contrasted with a motor baseline task. Specifically, we expected skill acquisition to be associated with activation of the SPL and IPS, a pattern consistent with previous studies on motor sequence learning, and knot-tying specifically (Cross et al., 2017; Mason and Just, 2020; Morgan et al., 2015). (ii) As a consequence of our participants gaining knot-tying experience as they went through the experimental tasks, we expected some transfer of knowledge to be visible when comparing the first knot the participant learned with the last. Because certain elements of the knot-tying sequences for the different knots are visually and motorically similar (Apšvalka et al., 2018), we expected to see decreased activation in DLPFC for the last knot. The reason being that the participants would be able to reuse some of the information they acquired in learning the previous knots, leading to decreased cognitive demands (Vogt et al., 2007). If this generalization of knowledge made the acquisition of later knots more efficient, we also expected to see a shift in activation from the precentral gyrus (PreCG) for the first knots, to the postcentral gyrus (PoCG) for the later knots. Increased reliance on sensorimotor areas after practice has been shown in the acquisition of simple stone toolmaking skills and might indicate a transition from active attention to procedural memory (Putt et al., 2019). (iii) Lastly, we hypothesized that in the absence of any other selective pressures, the accumulation of behavioral changes along the chain would make the information presented to later generations of participants easier to replicate, compared to what was presented to earlier generations. If this was the case, there should be a higher number of incorrectly replicated knots in earlier generations than in later ones. Because of this, we expect to see a pattern of neural activation similar to that of experience, with decreased DLPFC and PreCG activation and increased PoCG activation in later generations compared to earlier ones.

## 3 Methods

### 3.1 Participants

A total of 24 healthy participants (14 female, 10 male, mean age 22.4) were recruited for the study via postings on notice boards at the campuses of the University of Bergen. Those interested in participating in the study filled out an online questionnaire used to determine their eligibility to participate and assign them to an experimental group. Screening was based on prior experience with knot-tying through activities such as boating, climbing, or boy/girl scouts, handedness [self-reported and confirmed by a 17-item handedness questionnaire (Raczkowski et al., 1974)], gender, and physical, medical or psychological factors that could affect neurological function. Only right-handed individuals reporting no in-depth experience with knot-tying were asked to participate.

Participants were assigned to one of three chains (8 participants per chain), based on their gender and handedness as follows: Chain 1 consisted of right-handed female participants only, Chain 2 consisted of right-handed male participants only, and Chain 3 alternated between left- and right-handed participants of either gender. Gender was kept consistent within Chain 1 and 2 to avoid potential influences of own-gender imitation biases (Cracco et al., 2018; Bussey and Bandura, 1984; Losin et al., 2012; Perry and Bussey, 1979; Shutts et al., 2010). To reduce potential confounding factors associated with handedness and brain lateralization during motor observation and imitation (Crotti et al., 2022) in this initial stage, the present paper is based on the data from Chain 1 (mean age 21.4) and Chain 2 (mean age 25.4). Which generation, or position within the chain, a participant was assigned to was determined by the order in which they participated and was a result of the participants’ availability.

The Regional Committees for Medical and Health Research Ethics (REK) in Norway approved the project ahead of participant recruitment (#351425). Written informed consent was obtained from all participants before the fMRI procedure.

### 3.2 Materials

#### 3.2.1 Instruments

Functional magnetic resonance images were collected using a T2*-weighted echo-planar imaging (EPI) sequence on a 3T GE Discovery (MR750) MRI scanner with a 32-channel head coil, located in the department of radiology at the Haukeland University Hospital in Bergen, Norway.

#### 3.2.2 Stimuli

The experimental stimuli consisted of six 30-s, silent video demonstrations: one for each of the five knots and one for the control condition. The videos showed the demonstrator’s hands on a plain background, shot from a 90°, overhead angle, giving the participants a first-person view to facilitate imitative learning (Watanabe et al., 2013; Uomini and Lawson, 2017). The videos were shot on a Sony A65 camera approximately 75 cm from the surface of the table. All knots were tied three times, and the camera filmed continuously until all knots had been tied. The video was cut down to create separate videoclips for each knot (in DaVinci Resolve 18.1.17). The videoclips used the last repetition of each knot, since the knot-tying usually was smoother and less hesitant after having gone through the steps a few times. All video clips were zoomed in to eliminate excess space around the hands and slightly slowed down to last for the full 30 s.

The knots used in the experiment were obtained from Animatedknots.com and classified as basic. Knots that the participants were likely to already be familiar with, such as the overhand knot, as well as knots requiring supplies other than the rope itself, were excluded. The five knots used in the experiment (see Figure 1) were the figure eight knot (A), the noose knot (B), the sheet bend (C), the slip knot (D), and the square knot (E). For the knots joining two rope-ends (the sheet bend and the square knot), both ends of a single rope were used. Initial piloting of the selected knots confirmed that they could be reliably learned within 2 min or less by knot-naïve individuals. In the control task, the demonstrator ran their hands along the rope from one end to the other and back again. This condition was intended to serve as a contrast to the knot-tying task, to isolate the activation associated to learning the steps of tying the knots. Several 60 cm pieces of braided, white polyester rope (5 mm) were used, both during the experiment and in the video demonstrations. Stimuli were presented and synced to functional data using NordicAktiva (v1.3.0) (NordicNeuroLab, 2020).

### 3.3 Procedure

The experiment consisted of two phases: the learning phase and the teaching phase. In the learning phase, participants viewed the video demonstrations and practiced tying the knots. This took place within the MRI scanner. In the subsequent teaching phase, participants demonstrated how to tie the knots they had just learned. The demonstrations took place in the lab shortly after completing the first phase. After completing both phases of the experiment, participants were debriefed and given a 300 NOK gift card as compensation for their time.

#### 3.3.1 Learning phase and fMRI

Upon arriving participants were walked through the experimental procedure to ensure they understood the task they were to perform. While in the MRI scanner, participants were guided through the experiment via instructions and videos displayed on a screen located near their feet. A set of mirrors mounted on the head coil was adjusted for each participant to ensure they could see both the screen and their own hands (while tying the knots), without needing to adjust their position. Participants were also instructed to always keep their elbows resting on the MRI table at either side of their body, in order to minimize movement during knot-tying.

The scanning session followed a block design with separate runs for each of the experimental stimuli (total of six runs). Each run consisted of six blocks, meaning that the participant was presented with the same knot six times before moving on to the next one. The structure of all blocks was identical with fixed timing for stimuli presentation. Before the start of each run six ropes were placed across the participants’ hips, and they were instructed to take one rope at a time to use for knot-tying practice. The blocks started with the presentation of the stimulus, where the participants watched a 30-s knot-tying video demonstration (observation condition). When the video ended, white text on a black background instructed the participants to recreate tying the knot they had just watched (practice condition). After 30 s, the text on the screen was replaced by a fixation cross that signaled the participants to discard the rope and wait for the next block to start. This resting period lasted for 15 s. Each run had a total duration of 7.5 min, giving the participant 3 min of physical practice per knot. The first generation of participants in each chain watched video demonstrations recorded by the researchers. From then on, the videos shown to each new participant were the recordings of the individual immediately prior to them in the chain.

#### 3.3.2 Teaching phase and videotaping

After the fMRI session, participants were led into another room to record the video demonstration for the next participant in their chain. During the demonstration, participants were seated at a desk and instructed to keep their hands in the center of a black mat on the table in front of them. This made sure that their hands were in frame and there was a good contrast between the background and their hands with the rope while tying the knots. Participants were shown the video of each knot once before starting their demonstration (the same videos as they were shown in the scanner). They were asked to tie the knots as if they were showing someone who did not know how to tie them. Participants were not asked to verbally explain the knot-tying process as they were demonstrating, since no sound would be included in the final video. They demonstrated each knot three times to guarantee there was usable footage. To avoid mislabeling, all knots were demonstrated in a fixed order and sorted alphabetically in this order: control task, figure eight knot, noose knot, sheet bend, slip knot, and square knot. The video camera was recording nonstop from first to last trial. The videos were later edited to fit the 30-s time slot in the fMRI paradigm.

One participant (generation 3, Chain 2) was excluded during data collection due to a lack of understanding of the experimental task, and failure to replicate the control condition. Their data was excluded from all analyses and no video recordings were produced. Instead, the videos made by the previous participant (generation 2, Chain 2) were used again in the following generation.

### 3.4 Behavioral performance assessment

Participant performance was determined by evaluating how well each participant’s video demonstrations matched the knot-tying of the corresponding video of the participant prior to them in the chain. The reason for measuring performance relative to the previous participant rather than to the original knots was that we expected the knots to be altered due to the repeated transmission. If compared to the original stimuli, one participant making a mistake would lead to all of the following participants being marked as having tied the knot incorrectly, even if they perfectly replicated the information presented to them. Rating the knot-tying relative to the previous participant also allowed us to see if the changes that accumulated along the chain made the altered knots easier to learn, or if the amount of replication mistakes remained constant. Knot-tying performance was given a score from 1 to 5 according to the following criteria: 1 = none of the knot-tying steps were correct, 2 = less than half of the steps were correct, 3 = half or more of the steps were correct, 4 = the knot was functionally correct, but the knot-tying process was somewhat altered or there was some esthetic deviations in the final knot, 5 = the knot was tied exactly as demonstrated. A one-way ANOVA followed by a Tukey post-hoc test was performed to check for performance differences related to the specific knot, chain, knot order, and gender.

To analyze how the knot-tying process changed along the chain, the number of steps was counted for each of the knots for all participants. A one-way repeated measures ANOVA was used to assess if there was an effect of generation on the number of steps in the knot-tying process.

### 3.5 fMRI data acquisition and processing

Three hundred whole-brain volumes were collected for each of the five knots and the control (repetition time (TR) = 1,500 ms, echo time (TE) = 33 ms, flip angle = 80°, field of view (FOV) = 230 mm, matrix size = 128 × 128, number of slices = 46, slice thickness = 3 mm, inter-slice gap = 0 mm, voxel size = 1.80 × 1.80 × 3.0 mm^3^). Additionally, high-resolution structural images were acquired using a T1-weighted magnetization-prepared rapid gradient echo (MP-RAGE) sequence (TR = 2,992.9 ms, TE = 2.82 ms, flip angle = 8°, FOV = 256 mm, matrix = 512 × 512, number of slices = 196, slice thickness = 1 mm, inter-slice gap = 0 mm).

The first four scans of each run were discarded prior to preprocessing to allow for T1 equilibrium effects. Preprocessing and statistical analyses were done using SPM12 (Wellcome Trust Centre for Neuroimaging, 2020) running on MATLAB version R2021a (MathWorks, 2021). During preprocessing, all images were realigned to the first scan of the first run and unwarped to correct for motion artifacts and field inhomogeneities. Following this, images were coregistered to the T1 image, the T1 image was segmented and normalized to the MNI template, and the derived normalization parameter was applied to the fMRI scans. Lastly, spatial smoothing with a 6 mm (FWHM) Gaussian smoothing kernel was applied to all volumes to enhance the signal-to-noise ratio.

A first-level, general linear model analysis was performed individually on data from each participant. A standard hemodynamic response function was used to model participant responses, with onsets aligned to the start of the video demonstration (observation condition) and the knot-tying practice (practice condition). The cut-off for the high-pass filter was set to 158 s to avoid filtering out task-related signals. Separate, whole brain contrast images were created for each knot separately, as well as one for all the knots combined, contrasting knot-tying with the control. A set of three images was produced for each contrast, one isolating the neural activity for when the participants were watching the video demonstration (observation condition), one isolating the neural activity while the participant practiced tying the knot (practice condition), and one that combined both the observation and practice conditions (combined condition).

Second-level analyses were applied to all participants as one group (*n* = 15), as well as to Chain 1 (all female, *n* = 8) and Chain 2 (all male, *n* = 7) separately. Three sets of analyses were performed. First, a one-sample *t*-test was used to compare activation during knot-tying to the activation during the control task. This was done to evaluate the effect of learning the knots. Second, the effect of knot-tying experience was evaluated by running a related measures, two-sample *t*-test comparing the single knot contrast images of the first knot the participant learned to the last knot they learned. Third, the effect of repeated skill transmission was investigated by comparing the first three generations of participants from both chains to the last three generations again using a related measures, two-sample *t*-test. All analyses used a voxel-wise *p* value adjustment of 0.001 (uncorrected), and a cluster-wise threshold of *p* = 0.005 (FWE-corrected).

## 4 Results

### 4.1 Behavioral outcomes

**Figure 2:**
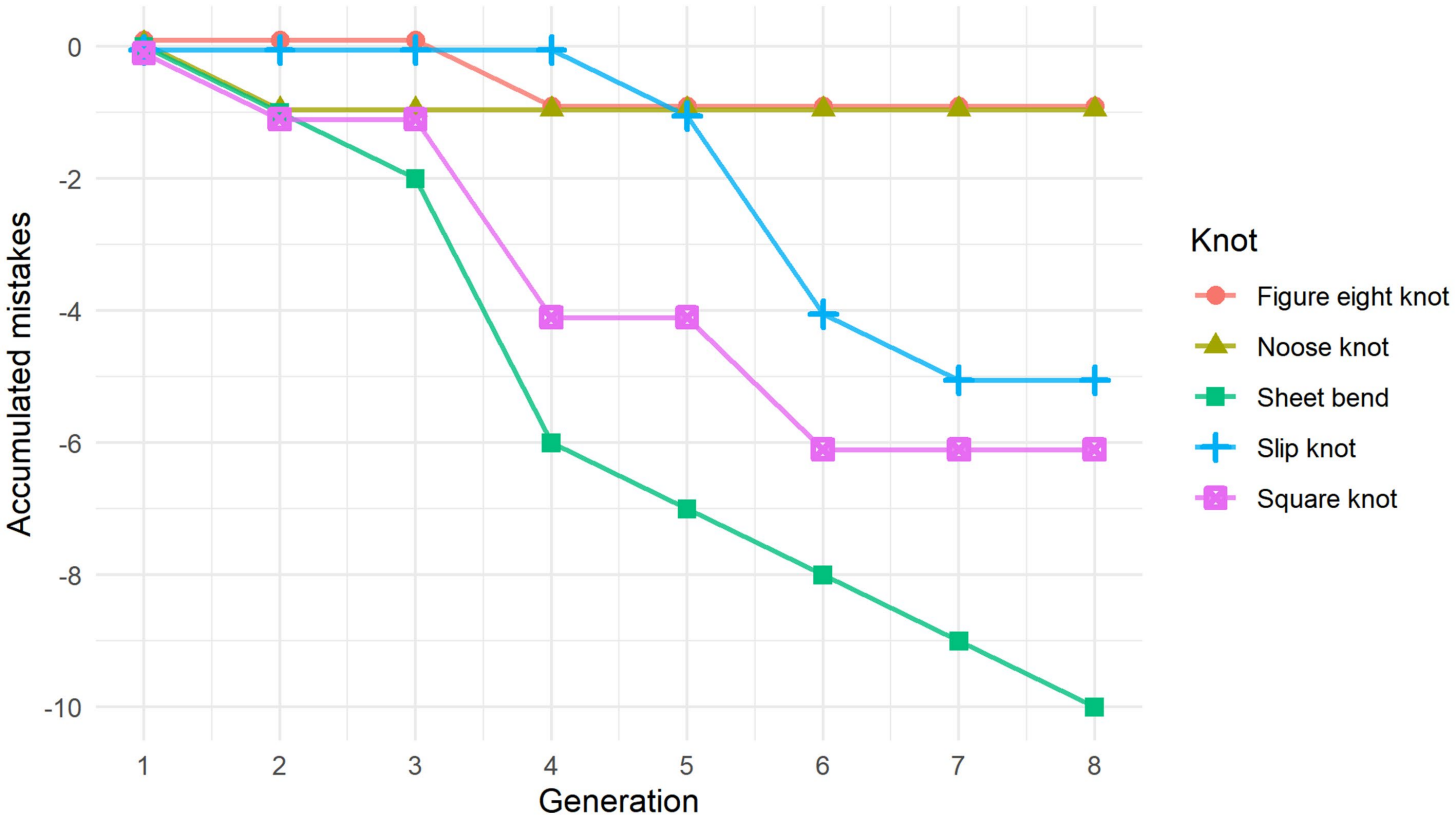
Line graph showing the accumulation of errors made to the individual knots in relation to the original stimuli. Curve-steepness reflects the knot’s similarity to that of the previous generation, with a horizontal line indicating a perfect replication of the knot stimuli they were shown.

Figure 2 shows how changes accumulated with generational transmission based on performance scores aggregated across chains. A one-way ANOVA was performed to check for performance differences related to the specific knot, chain, knot order, and gender. The results showed a significant difference in performance dependent on which knot was being tied (*F*(4) = 3.589, *p* < 0.05). A Tukey *post hoc* test revealed that participants performed significantly better on the figure eight knot (*M* = 5.00) than the sheet bend (*M* = 3.73) (*p* < 0.05). Aside from this, no other knots showed significant differences in performance. We also did not find any significant interaction effects between knot-tying performance and any of the other factors, indicating that performance was not affected by participant gender, their position within the chain, or the order in which they learned the knots.

There was some variation when it came to the number of steps it took to tie the different knots for individual participants. This was due both to replication errors and changes in knot-tying technique where some steps were combined. The repeated measures one-way ANOVA used to assess the effect of generation on the number of steps in the knot-tying process did not reveal any significant differences. However, when we looked at the average amount of knot-tying steps for each generation, there seemed to be a trend toward a decrease among the later generations, so the lack of statistical significance might be due to the small dataset.

In both chains all but one knot had accumulated at least one error. Visual inspection of the knots revealed that replication mistakes made along the chain led to an altered overall shape of the knot half of the time. For example, the sheet bend originally ties together two rope ends, but in both chains a mistake to the knot moved it to the middle of the rope. On the other hand, the figure eight knot and the noose knot retained the overall shape for the entire chain, even when replication mistakes were made along the way.

### 4.2 Functional data

#### 4.2.1 Effect of skill acquisition

To assess the cognitive aspect of learning the knot-tying sequence, we compared knot-tying to the control task. No clusters of neural activation emerged in the control > knot-tying contrast; therefore, all reported results are based on the knot-tying > control contrast.

**Figure 3:**
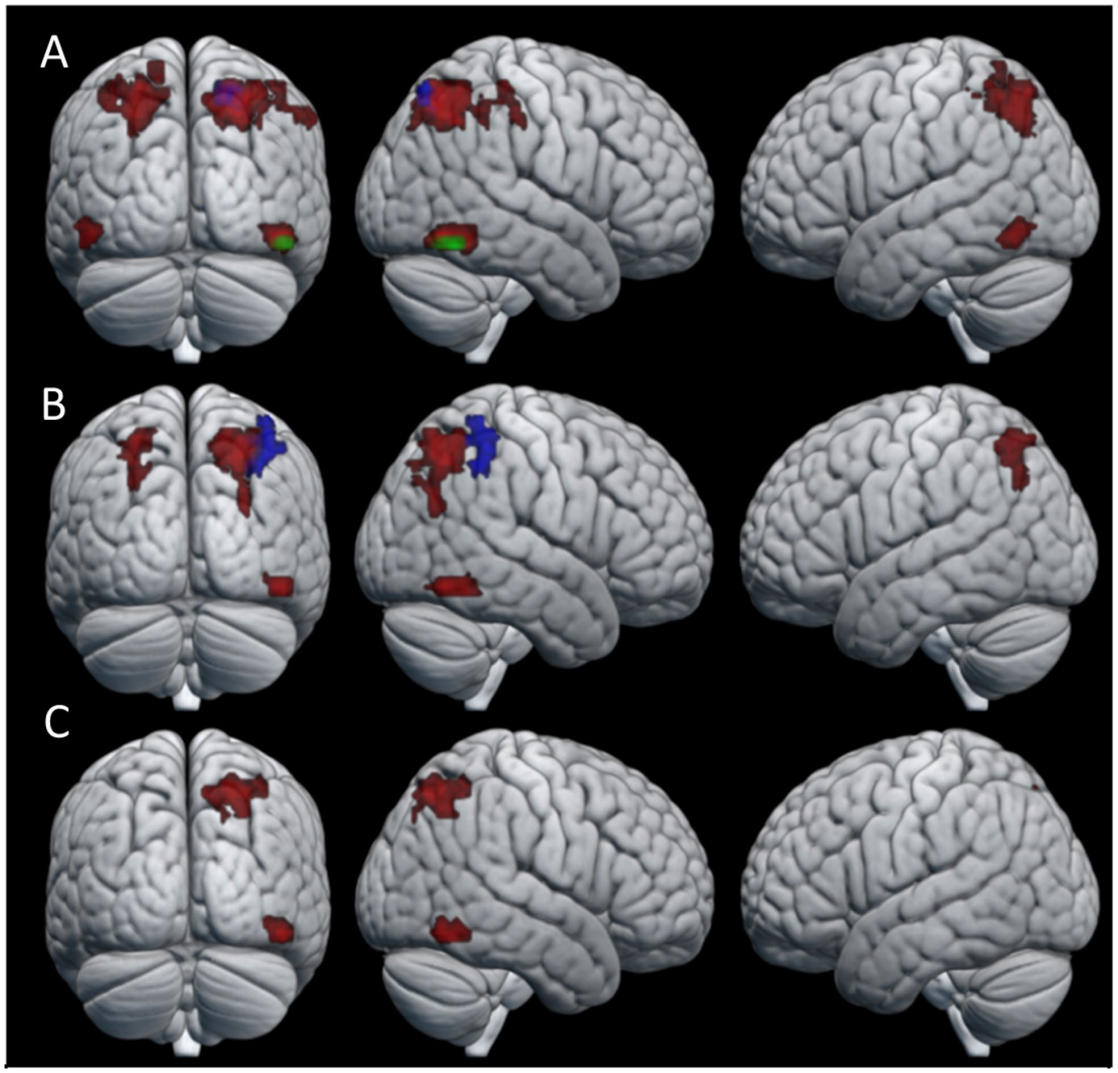
Areas of neural activity for the knot-tying > control contrast in the **(A)** combined condition **(B)** observation condition, and **(C)** practice condition. Red areas show overall activation, green areas show Chain 1 activation, and blue areas show Chain 2 activation.

We identified a total of four clusters that were significantly more active during knot-tying compared to the control task for the combined condition (Table 1 for details). These clusters were located in the fusiform gyrus (FG) bilaterally, the superior parietal lobule (SPL) of the right hemisphere, and the IPS of the left hemisphere (see Figure 3A). When looking at the chains separately, only the cluster in the right FG survived in Chain 1. In Chain 2, only the cluster in the right SPL survived. However, a two-sample *t*-test directly comparing Chain 1 and Chain 2 did not reveal any significant differences in activation between the two chains. The differences in activation between the two chains might therefore be due to the small sample size. Panel A of Figure 3 shows the location of the clusters for all participants, and each chain separately.

**Table 1:** Areas of increased activity in the knot-tying>control contrast in the combined condition.

Region	Cytoarchitectonic area	Cluster size	*t*-value	*z*-score	*x*	*y*	*z*
All participants							
R fusiform gyrus	FG2	108	11.01	5.553	48	−64	−13
L fusiform gyrus	FG2	54	5.1761	3.807	−51	−64	−13
R superior parietal lobule^*^	7A	345	7.5352	4.69	21	−67	50
L intraparietal sulcus^*^	hIP6	251	5.8078	4.078	−24	−67	56
Chain 1							
R fusiform gyrus	FG4	34	8.3882	3.986	45	−55	−16
Chain 2							
R superior parietal lobule	7P	38	12.85	4.349	18	−70	56

Results of a one sample t-test of the knot-tying > control contrast for the combined condition.

Voxel-wise significance threshold was *p* < 0.001. Only clusters that survived FWE cluster-correction of *p* < 0.05 are reported. Coordinates indicate peak voxel location in MNI space.

* Indicates clusters with a significance level of *p*(FEW) < 0.001.

R, right; L, left.

The pattern of activation in the observation condition is fairly similar to that of the combined condition (see Table 2 for details). We found significant differences between the knot-tying and the control task for all participants in the right FG and bilateral IPS. When looking at the chains separately, Chain 2 revealed one cluster in the right SPL. The activation associated with the observation condition is visualized in Panel B of Figure 3.

**Table 2:** Areas of increased activity for the knot-tying > control contrast in the observation condition.

Region	Cytoarchitectonic area	Cluster size	*t*-value	*z*-score	*x*	*y*	*z*
All participants							
R fusiform gyrus	FG2	76	6.535	4.356	45	−61	−13
R intraparietal sulcus^*^	hIP3	224	6.244	4.249	24	−61	47
L intraparietal sulcus^*^	hIP6	105	5.428	3.919	−24	−73	56
Chain 2							
R superior parietal lobule^*^	7PC	82	11.935	4.254	39	−46	62

Results of a one sample t-test of the knot-tying > control contrast for the observation condition.

Voxel-wise significance threshold was *p* < 0.001. Only clusters that survived FWE cluster-correction of *p* < 0.05 are reported. Coordinates indicate peak voxel location in MNI space.

* Indicates clusters with a significance level of *p*(FEW) < 0.001.

R, right; L, left.

In the practice condition, we found significant activation in the SPL, and the FG, both in the right hemisphere (see Table 3 for details). Panel C of Figure 3 shows where the activation associated with this condition was located. This condition did not reveal any significant differences in neural activation when looking at the chains separately.

**Table 3:** Areas of increased activity for the knot-tying > control contrast in the practice condition.

Region	Cytoarchitectonic area	Cluster size	*t*-value	*z*-score	*x*	*y*	*z*
All participants							
R fusiform gyrus	FG2	65	5.951	48	48	−64	−13
R superior parietal lobule^*^	7A	161	6.417	30	30	−70	53

Results of a one sample t-test of the knot-tying > control contrast for the practice condition.

Voxel-wise significance threshold was *p* < 0.001. Only clusters that survived FWE cluster-correction of *p* < 0.05 are reported. Coordinates indicate peak voxel location in MNI space.

* Indicates clusters with a significance level of *p*(FEW) < 0.001.

R, right.

#### 4.2.2 Effect of knot-tying experience

**Figure 4:**
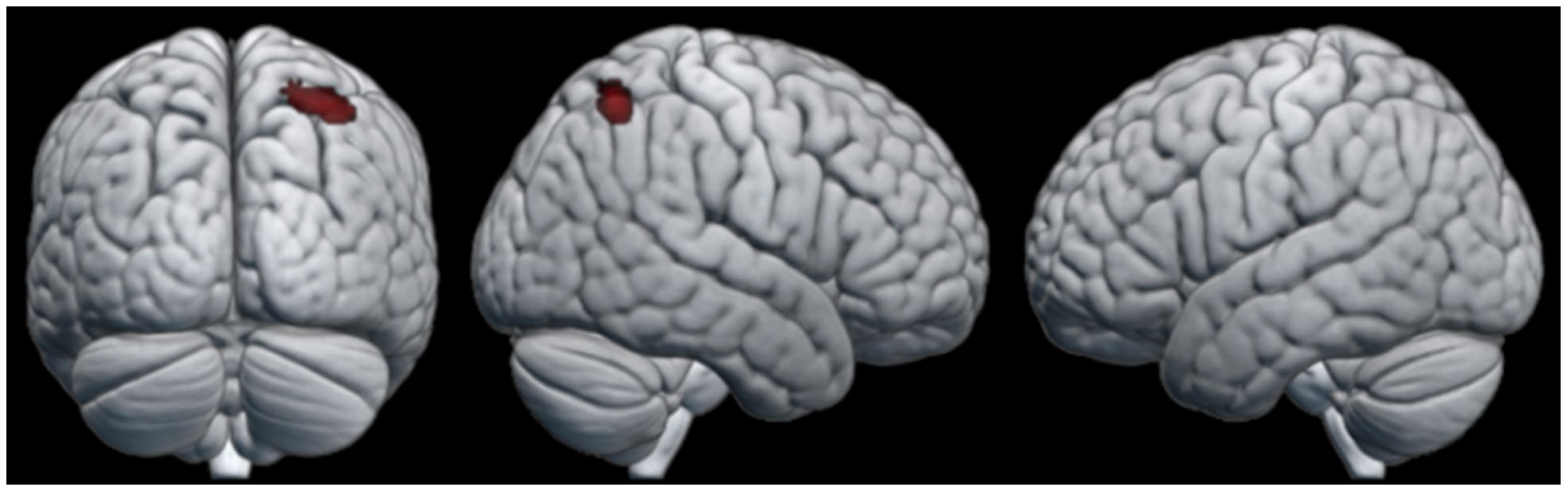
Areas of neural activity for the last knot > first knot contrast in the observation condition.

To look at the effect of the gaining knot-tying experience during the course of the experiment, a comparison of the first and last knot the participants learned was performed. No clusters of neural activation reached statistical significance for the first knot > last knot contrast. In the last knot > first knot contrast, however, the observation condition produced a cluster of neural activation that reached statistical significance. This cluster was located in the SPL of the right hemisphere (see Table 4 for details). The pattern of neural activation of this contrast is visualized in Figure 4.

**Table 4:** Areas of increased activity for the last knot > first knot contrast in the observation condition.

Region	Cytoarchitectonic area	Cluster size	*t*-value	*z*-score	*x*	*y*	*z*
All participants							
R superior parietal lobule	7A	71	5.207	4.318	33	−55	56

Results of a related measures, two sample t-test of the last knot > first knot contrast for the observation condition.

Voxel-wise significance threshold was *p* < 0.001. Only clusters that survived FWE cluster-correction of *p* < 0.05 are reported. Coordinates indicate peak voxel location in MNI space.

R, right.

#### 4.2.3 Effect of repeated skill transmission

**Figure 5:**
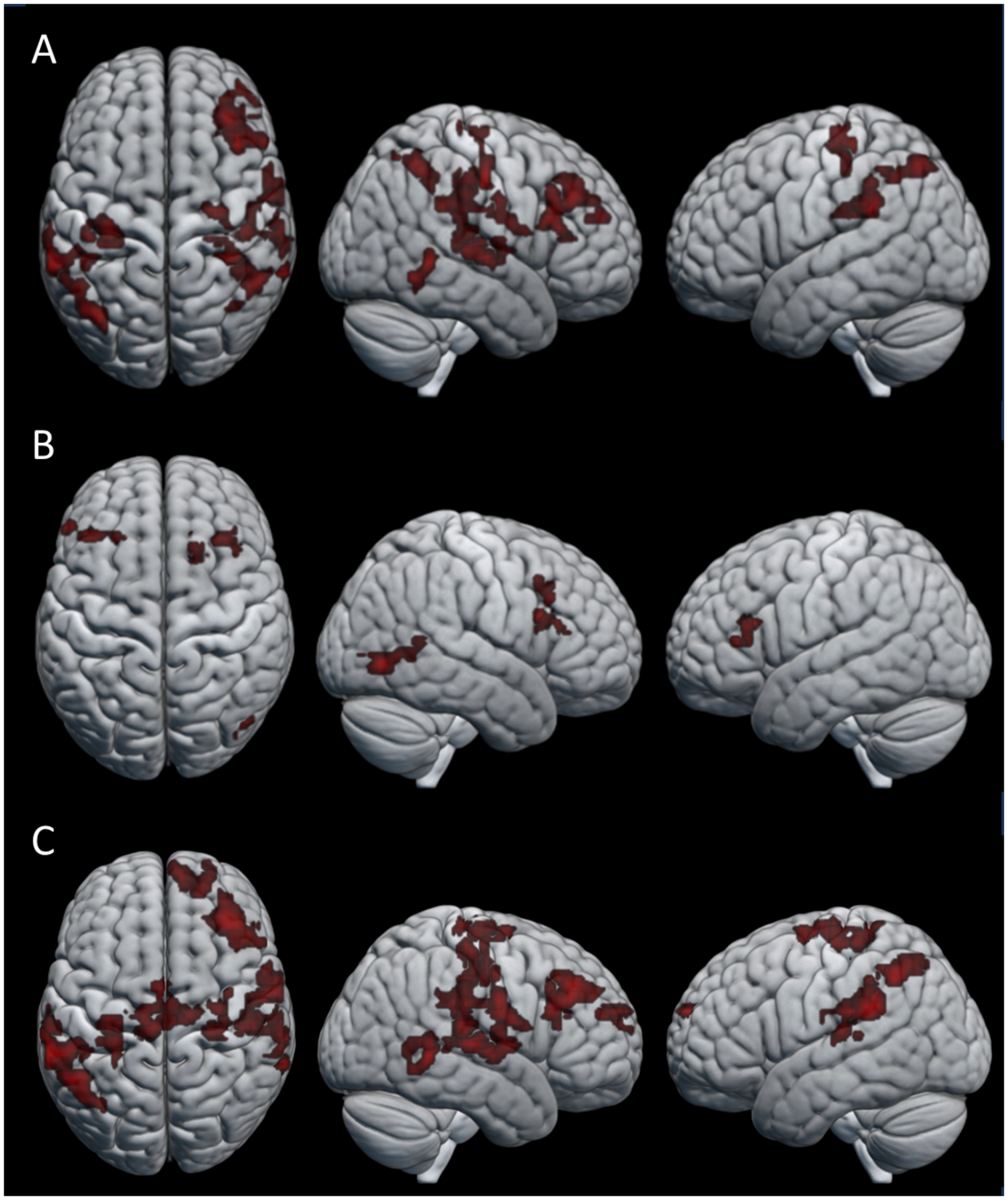
Areas of activation for the last generations > first generations contrast in the **(A)** combined condition, **(B)** observation condition, and **(C)** practice condition.

To look at the effect of the repeated transmission of knot-tying skills, we compared the single knot images of participants in the first three generations (six participants) to those that made up the last three generations (six participants) of both chains. Only the last generations > first generations contrast yielded significant differences in neural activation. Figure 5 shows the pattern of activation for (A) the combined condition, (B) observation condition, and (C) practice condition (see Table 5 for details). The combined and practice conditions revealed similar patterns of neural activation, with clusters located in the right middle frontal gyrus (MFG), the precentral gyrus bilaterally (PreCG), left angular gyrus (AG), right middle temporal gyrus (MTG), and left SMG. A cluster in the right AG was unique to the combined condition, and the practice condition showed additional activation in the right medial superior frontal gyrus (mSFG), the right planum temporale (PT), and left middle cingulate gyrus (MCG). The pattern of neural activation for the observation condition did however differ from this, with only three clusters of significant activation. These clusters were located in the right FG and the MFG bilaterally.

**Table 5:** Areas of increased activity for the last generations > first generations contrast in the combined, observation and practice condition.

Region	Cytoarchitectonic area	Cluster size	*t*-value	*z*-score	*x*	*y*	*z*
Combined							
R middle frontal gyrus^*^	IFS4	232	5.906	5.204	39	20	23
R precentral gyrus	3a	73	5.516	4.925	30	−16	41
L precentral gyrus	4a	93	4.569	4.205	−33	−22	65
L supramarginal gyrus	PF	105	6.229	5.428	−63	−40	29
R middle temporal gyrus	21	66	5.675	5.04	63	−46	−1
R angular gyrus^*^	Pga	458	5.729	5.079	51	−52	50
L angular gyrus	Pga	84	5.504	4.916	−36	−67	50
Observation							
R middle frontal gyrus	44	98	6.319	5.489	24	17	23
L middle frontal gyrus	44	63	5.737	5.084	−21	23	20
R fusiform gyrus	FG1	92	5.835	5.154	30	−70	−4
Practice							
R medial superior frontal gyrus	9	70	6.172	5.389	9	59	23
R middle frontal gyrus^*^	46	244	5.747	5.092	39	32	35
R precentral gyrus	4	94	4.881	4.449	30	−16	41
L precentral gyrus	4	92	4.771	4.364	−27	−22	59
R planum temporale^*^	22	540	5.803	5.132	57	−19	5
L middle cingulate gyrus^*^	24, 32	298	4.787	4.376	−12	−28	32
R middle temporal gyrus	21	110	5.890	5.193	66	−46	−1
L supramarginal gyrus^*^	40	205	6.667	5.721	−60	−40	26
L angular gyrus	39	123	5.493	4.909	−45	−52	41

Results of a related measures, two sample t-test of the later participant generations > early participant generations contrast for the combined condition, the observation condition, and the practice condition.

Voxel-wise significance threshold was *p* < 0.001. Only clusters that survived FWE cluster-correction of *p* < 0.05 are reported. Coordinates indicate peak voxel location in MNI space.

* Indicates clusters with a significance level of *p*(FEW) < 0.001.

R, right; L, left.

## 5 Discussion

### 5.1 Acquisition of knot tying skills

Overall, most participants managed to correctly replicate four out of the five knot stimuli they were presented with, indicating that the task difficulty was not too high, and participants generally managed to learn the knots. The highest number of incorrectly replicated knots for an individual participant was three, and happened for only three out of the 15 participants included in this study. To identify the neural regions involved in the acquisition of knot-tying skill, we compared knot-tying to a control task involving the mere manipulation of a rope. We found a distinctive pattern of activation for knot-tying suggesting this was likely related to learning the task. The results revealed that during the acquisition of knot-tying skills, besides the activation of the FG, there was particular activation in clusters covering portions of the SPL and IPS in both hemispheres. These findings are in line with previous research on learning to knap simple stone tools (Stout and Chaminade, 2007; Stout et al., 2011) and knot-tying (Cross et al., 2017). The FG has been associated with the processing of complex visual information (Lorenz et al., 2017). In this context it might have contributed to recognizing visual patterns associated with the different knots to guide motor learning. The IPS has been implicated in visual analysis during object manipulation and tool handling (Stout and Chaminade, 2007). The SPL is associated with spatial awareness and the coordination of motor actions, which are essential for tasks requiring precise manual dexterity (Cross et al., 2017; Stout et al., 2008). The activation of the SPL then suggests that knowledge of objects and actions and the guiding of action in space are relevant elements for learning how to tie a knot. Finally, both the SPL and IPS have been found to play a role in attention control, particularly in focusing and orienting attention (Rueda, 2018), which are key processes involved in learning a novel task. This agrees with previous interpretations of the human capacity for skill learning and execution being more dependent on sensorimotor capabilities, rather than executive capabilities (Stout and Chaminade, 2007).

### 5.2 Effect of gaining experience

The effect of gaining knot-tying experience through the course of the experiment was investigated by comparing each participant’s last knot to their first. When looking at behavioral performance there was no significant differences between knots related to the order in which participants learned them. The comparison of the functional data revealed a single cluster of significant activation in the right SPL extending into the IPS, when participants watched the tying of the last knot. This result partially coincides with the knot-tying experiment by Cross et al. (2017), which showed that observational as well as physical experience with tying specific knots was associated with activation of the right IPS and the right dorsal premotor cortex. However, the same study reported left SPL activation related to physical experience specifically, an area which has been previously linked to the creation of mental representations (Mason and Just, 2020). The fact that our study did not find activation of the left SPL despite increased experience may be due to ceiling effects since we chose the knots mostly based on them being simple enough to be learned in a short amount of time. If this is the case the lack of significant activation is likely due to overlap in neural activation for both the first and last knot. Alternatively, the short duration of our sessions might not have allowed enough time for the participants to fully master the observed knots and establish the corresponding mental representations. We view this explanation as less likely since no statistical difference in knot-tying performance was found for knots based on the order they were learned.

### 5.3 Repeated transmission of skill

As we had expected, most of the final knots and the knot-tying techniques became different from the original ones. However, the degree to which the knots differed from the original varied (see Figure 2). Half of the knots where at least one error occurred during transmission had their general shape altered. There was also an overall trend toward decreasing the number of steps in the knot-tying process when comparing the original stimuli to the knots demonstrated by the last participant, though this did not reach statistical significance. The lack of statistical significance might be due to the low number of steps in the original stimuli. Another possible explanation is that the sample size was too small.

Changes also occurred in the knots that survived for the entire transmission chain. One such example is the noose knot in Chain 1. This knot stayed the same in terms of function, but the process of tying the knot had been shortened. A possible explanation is that the original technique was perceived as difficult and for that reason, each generation collectively contributed to its simplification, without altering the final product. A simple technique would have been easier to learn and pass down to future generations, ultimately becoming more time efficient. It is also possible that the alteration resulted from the constraints of the experiment, since the participants had to learn to tie the knots while lying down in the fMRI scanner, instead of sitting as in the demonstration videos. In other words, the simplification of the technique may be attributed to coping with the movement restrictions of the arms and the head.

Even though most of the final knots were different from the original stimuli, at the end of the chain all but one of them were still stable knots that retained their shape after being let go of. This might indicate that participants were more focused on replicating the steps of the knot-tying process, rather than the end product, which is similar to what is seen in cultural transmission tasks requiring high fidelity copying (e.g., know-how) (Andersson and Tennie, 2023). It also indicates that even simple knots are at least somewhat cognitively opaque (Wasielewski, 2014), and that the acquisition of knot tying skills relies on social learning (Kaaronen et al., 2024).

To evaluate how the accumulation of behavioral changes along the transmission chains influences the cognitive demands of skill acquisition, we compared the last three generations of participants with the first three generations. Our findings revealed distinctive clusters of neural activation in a number of frontal, parietal and temporal regions among the later generations of participants linked to the physical practice of knot-tying. Frontal lobe activation had clusters centered in the PreCG bilaterally, covering areas of both primary motor and somatosensory cortex, linked to executing voluntary movements and action observation and the MFG and mSFG, related to executive functions and self-observation. Parietal lobe activation was found in the AG and SMG, linked to the translation of visual information to motor action and proprioception respectively; Temporal lobe activation was found in the right PT, involved with object recognition (Nilakantan et al., 2017); and the MTG, associated with semantic memory (Xu et al., 2015; Stout et al., 2011). In contrast to our expectations the pattern of activation is broadly consistent with studies looking at the production of complex stone tools, indicating increased cognitive demands for effective visuomotor coordination and working memory (Hecht et al., 2015; Putt et al., 2017; Putt et al., 2019; Stout and Chaminade, 2007; Stout et al., 2008).

Both the MFG and mSFG activation fall within the functional area of the DLPFC. Putt et al. (2019) linked increased DLPFC activity to the learning of motor task with higher demands for attention and working memory, such as learning to knap complex stone tools. In contrast, learning to knap simple stone tools did not involve increased DLPFC activation, but rather relied on premotor cortex activation that shifted to sensorimotor areas as participants gained experience with the flint knapping task. This was explained by participants starting to anticipate upcoming actions as they became more familiar with knapping and the process was automated. Our finding that the DLPFC was significantly more active among the later generations compared with the first generations is puzzling. One possible explanation might be that the changes in the knots down the chain led to knot-tying processes that became less intuitive. A less intuitive process might have required more cognitive effort to remember, even with fewer steps, compared to the earlier generations which learned the knots before they had been altered by transmission.

In agreement with our expectations, the acquisition of knot-tying skill yielded activity in the IPS and SPL. However, it additionally showed activation in the FG, which relates to the processing of visual information and visual recognition. This pattern of activation highlights the importance of sensorimotor integration and high-level visual functions such as object recognition during observational learning and physical practice of a sequential motor task. When looking at the effect of knot-tying experience the right SPL was significantly more active when participants learned the last knot compared to the first knot, but only during observation. This area is associated with the processing of tactile information, spatial orientation, attention control, and working memory, and might reflect increased effort of keeping the information of the different knots separate from each other. Finally, the effect of repeated transmission was related to increased activation of several frontal, temporal and parietal structures. Contrary to our expectations the activation of DLPFC during physical knot-tying practice for participants in later generations compared to earlier generations indicate an increased demand of executive functions. This might be due to the accumulation of behavioral changes along the chains making the knot-tying process less intuitive.

### 5.4 Limitations and future directions

Since this is a pilot study with a limited sample size we will not draw any definitive conclusions based on its findings. However, there are a number of interesting trends we think deserve further investigation. Prior to the study, we hypothesized that learning, experience, and repeated skill transmission would result in behavioral outcomes typical for transmission chain studies, and that these behavioral outcomes would be accompanied by distinct patterns of neural activation. Namely, we expected the knot-tying process to become more efficient and easier to learn. Our results, however, do not allow for a straightforward conclusion. As mentioned above, we found a trend toward the reduced number of steps in the operation of tying a knot along the chains, which can be attributed to efficiency, but it could also be interpreted as the loss of cultural information down the generations. Also as discussed, the activation of the DLPFC after repeated transmission of information may contradict our prediction, as it could be interpreted as an increase in cognitive effort.

There are several confounding factors related to the presentation of the knot-tying stimuli. Participants were asked to repeat the knots numerous times, first in the scanning session, then during the demonstration Additionally, participants were asked to demonstrate the knots in the second phase in an order that differed from the order in which they learned the knots. This coupled with the visual similarity between some of the original knot stimuli might have led to confusion and difficulty in distinguishing the knots from each other.

Other limiting factors of the present study are related to the use of fMRI. Even though this tool is highly conductive for collecting full brain images while not exposing participants to harmful radiation, it also comes with a range of drawbacks. Particularly for motor action studies like ours, the fact that the participant must be laying down in the machine limits the types of experiments afforded by this technology. Crucially, the use of fMRI severely restrains the possibilities for social interaction during learning. Even though observational learning is technically a form of social learning (Andersson and Tennie, 2023), the closest the participants came to social ‘interaction’ during our study was watching the demonstration videos. Vulnerability to motion artifacts further constrains the type of tasks one can do in the scanner. A promising avenue to explore in future research would be the use of functional near-infrared spectroscopy (fNIRS). Using fNIRS for a study like this would greatly increase its validity by allowing participants sitting next to each other to interact directly, while being less vulnerable to movement artifacts.

The present study also had limited statistical power. Although justified by this being an exploratory pilot study, the small sample size imposed by the costs and planning constraints of the fMRI scanner increases the risk of both type 1 and type 2 errors.

Finally, it is hard to determine whether the accumulated changes in the knots were due to random variation or some form of directional selection pressure. Participants watched videos of the knots and were requested to reproduce them, but the task made no requirements in terms of functionality. That is, participants were not given any information on the function or purpose of the knots, nor were the knots tested for any of this. In real life, the motivation for acquiring skills is to apply them in specific situations, so it may be that adding a performance task would affect participants’ motivation for high-fidelity social learning (Tennie et al., 2009). This is an interesting avenue that could be explored in future research. Also, the loss of information along the chains would have produced a qualitative difference between the information received by participants early in the chain compared to those later in the chain. This could be a confounding factor when comparing earlier generations with later generations, and subsequent studies should look for strategies to counteract this.

To sum up, while the results from this exploratory study are promising in regards of combining behavioral studies with neuroimaging to deepen our understanding of the underlying mechanisms of CCE, its limitations also point to ways in which future neurocognitive research in the topic can be improved. To begin with, we recommend that larger sample sizes should be aimed for, and mechanisms known to affect CCE, such as task complexity and teacher expertise, should be incorporated as potential influencing factors. It will, however, be theoretically essential to implement varying degrees of social interaction between participants.

## 6 Conclusion

This study was the first to combine the methodologies of transmission chain studies and brain imaging to contribute to our understanding of the cognitive mechanisms that support CCE. We found this approach to be both viable and useful. Despite the discussed limitations, we were able to identify changes in both neural activation patterns and behavioral outcomes related to learning, experience, and repeated skill transmission of a motor task, which can potentially help elucidate how human culture evolves and is maintained across generations. Finally, the findings from our study corroborate previous research on toolmaking and motor skill acquisition that may prove relevant for testing hypotheses about the evolution of human cognitive abilities.

## Data Availability

The datasets presented in this article are not readily available because of the conditions of the ethical approval for the project. Requests to access the datasets should be directed to Heidi.Ohrn@uib.no.

## References

[ref1] Andersson C. Tennie C. (2023). Zooming out the microscope on cumulative cultural evolution: ‘trajectory B’ from animal to human culture. Human. Soc. Sci. Commun. 10, 1–20. doi: 10.1057/s41599-023-01878-6, PMID: 39310270

[ref2] Apšvalka D. Cross E. S. Ramsey R. (2018). Observing action sequences elicits sequence-specific neural representations in frontoparietal brain regions. J. Neurosci. 38, 10114–10128. doi: 10.1523/JNEUROSCI.1597-18.2018, PMID: 30282731 PMC6596197

[ref3] Barham L. (2013). From hand to handle: the first industrial revolution. Oxford: Oxford University Press.

[ref4] Bar-Yosef Mayer D. E. Groman-Yaroslavski I. Bar-Yosef O. Hershkovitz I. Kampen-Hasday A. Vandermeersch B. . (2020). On holes and strings: earliest displays of human adornment in the middle Palaeolithic. PLoS One 15:e0234924. doi: 10.1371/journal.pone.0234924, PMID: 32640002 PMC7343129

[ref5] Boyd R. Richerson P. J. (1996). Why culture is common, but cultural evolution is rare. Proc. Br. Acad. 88, 77–93.

[ref6] Bussey K. Bandura A. (1984). Influence of gender constancy and social power on sex-linked modeling. J. Pers. Soc. Psychol. 47, 1292–1302. doi: 10.1037/0022-3514.47.6.1292, PMID: 6527216

[ref7] Caldwell C. A. (2020). Using experimental research designs to explore the scope of cumulative culture in humans and other animals. Top. Cogn. Sci. 12, 673–689. doi: 10.1111/tops.12391, PMID: 30375756 PMC7379729

[ref8] Caldwell C. A. Millen A. E. (2008a). Experimental models for testing hypotheses about cumulative cultural evolution. Evol. Hum. Behav. 29, 165–171. doi: 10.1016/j.evolhumbehav.2007.12.001

[ref9] Caldwell C. A. Millen A. E. (2008b). Studying cumulative cultural evolution in the laboratory. Philos. Trans. Roy. Soc. B Biol. Sci. 363, 3529–3539. doi: 10.1098/rstb.2008.0133, PMID: 18799419 PMC2607341

[ref10] Caldwell C. A. Millen A. E. (2009). Social learning mechanisms and cumulative cultural evolution: is imitation necessary? Psychol. Sci. 20, 1478–1483. doi: 10.1111/j.1467-9280.2009.02469.x, PMID: 19891752

[ref11] Caldwell C. A. Renner E. Atkinson M. (2018). Human teaching and cumulative cultural evolution. Rev. Philos. Psychol. 9, 751–770. doi: 10.1007/s13164-017-0346-3, PMID: 30595765 PMC6290649

[ref12] Cracco E. Bardi L. Desmet C. Genschow O. Rigoni D. De Coster L. . (2018). Automatic imitation: a meta-analysis. Psychol. Bull. 144, 453–500. doi: 10.1037/bul0000143, PMID: 29517262

[ref13] Cross E. S. Hamilton A. F. D. C. Cohen N. R. Grafton S. T. (2017). Learning to tie the knot: the acquisition of functional object representations by physical and observational experience. PLoS One 12:e0185044. doi: 10.1371/journal.pone.0185044, PMID: 29023463 PMC5638238

[ref14] Crotti M. Koschutnig K. Wriessnegger S. C. (2022). Handedness impacts the neural correlates of kinesthetic motor imagery and execution: a FMRI study. J. Neurosci. Res. 100, 798–826. doi: 10.1002/jnr.25003, PMID: 34981561 PMC9303560

[ref15] Currie A. Killin A. (2019). From things to thinking: cognitive archaeology. Mind Lang. 34, 263–279. doi: 10.1111/mila.12230

[ref16] Dean L. G. Kendal L. Schapiro S. J. Thierry B. Laland K. N. (2012). Identification of the social and cognitive processes underlying human cumulative culture. Science 335, 1114–1118. doi: 10.1126/science.1213969, PMID: 22383851 PMC4676561

[ref17] Fragaszy D. M. Biro D. Eshchar Y. Humle T. Izar P. Resende B. . (2013). The fourth dimension of tool use: temporally enduring artefacts aid primates learning to use tools. Philos. Trans. Roy. Soc. B Biol. Sci. 368:20120410. doi: 10.1098/rstb.2012.0410, PMID: 24101621 PMC4027420

[ref18] Fuster J. M. (2001). The prefrontal cortex—an update: time is of the essence. Neuron 30, 319–333. doi: 10.1016/S0896-6273(01)00285-9, PMID: 11394996

[ref19] Gerdes P. (2010). Tinhlèlò, interweaving art and mathematics: colourful circular basket trays from the south of Mozambique. Maputo and Lulu: Centro de Investigação Etnomatemática.

[ref20] Hardy B. L. Moncel M.-H. Kerfant C. Lebon M. Bellot-Gurlet L. Mélard N. (2020). Direct evidence of Neanderthal fibre technology and its cognitive and behavioral implications. Sci. Rep. 10:4889. doi: 10.1038/s41598-020-61839-w, PMID: 32273518 PMC7145842

[ref21] Hecht E. E. Gutman D. Khreisheh N. Taylor S. Kilner J. Faisal A. . (2015). Acquisition of Paleolithic toolmaking abilities involves structural remodeling to inferior frontoparietal regions. Brain Struct. Funct. 220, 2315–2331. doi: 10.1007/s00429-014-0789-6, PMID: 24859884

[ref22] Henshilwood C. d'Errico F. Vanhaeren M. Van Niekerk K. Jacobs Z. (2004). Middle stone age shell beads from South Africa. Science 304:404. doi: 10.1126/science.1095905, PMID: 15087540

[ref23] Hurcombe L. M. (2014). Perishable material culture in prehistory: investigating the missing majority. New York: Routledge.

[ref24] Kaaronen R. O. Henrich A. K. Manninen M. A. Walsh M. J. Wisher I. Eronen J. T. . (2024). The ties that bind: computational, cross-cultural analyses of knots reveal their cultural evolutionary history and significance. OSF Preprints. doi: 10.31219/osf.io/fw7s6

[ref25] Kuhn S. L. Clark A. E. (2014). “Stone tool technology” in The Oxford handbook of the archaeology and anthropology of hunter-gatherers. eds. Cummings V. Jordan P. Zvelebil M. (Oxford: Oxford Academic).

[ref26] Lorenz S. Weiner K. S. Caspers J. Mohlberg H. Schleicher A. Bludau S. . (2017). Two new cytoarchitectonic areas on the human mid-fusiform gyrus. Cereb. Cortex 27, 373–385. doi: 10.1093/cercor/bhv225, PMID: 26464475 PMC6248695

[ref27] Losin E. A. R. Iacoboni M. Martin A. Dapretto M. (2012). Own-gender imitation activates the brain’s reward circuitry. Soc. Cogn. Affect. Neurosci. 7, 804–810. doi: 10.1093/scan/nsr055, PMID: 22383803 PMC3475355

[ref28] Mason R. A. Just M. A. (2020). Neural representations of procedural knowledge. Psychol. Sci. 31, 729–740. doi: 10.1177/0956797620916806, PMID: 32396452

[ref8003] MathWorks. (2021). “Matlab”. R2021a. The MathWorks Inc. PC.

[ref29] Morgan T. J. Uomini N. T. Rendell L. E. Chouinard-Thuly L. Street S. E. Lewis H. M. . (2015). Experimental evidence for the co-evolution of hominin tool-making teaching and language. Nat. Commun. 6:6029. doi: 10.1038/ncomms7029, PMID: 25585382 PMC4338549

[ref30] Muthukrishna M. Shulman B. W. Vasilescu V. Henrich J. (2014). Sociality influences cultural complexity. Proc. R. Soc. B Biol. Sci. 281:20132511. doi: 10.1098/rspb.2013.2511, PMID: 24225461 PMC3843838

[ref31] Nilakantan A. S. Voss J. L. Weintraub S. Mesulam M. M. Rogalski E. J. (2017). Selective verbal recognition memory impairments are associated with atrophy of the language network in non-semantic variants of primary progressive aphasia. Neuropsychologia 100, 10–17. doi: 10.1016/j.neuropsychologia.2017.04.006, PMID: 28391035 PMC5501705

[ref8001] NordicNeuroLab. (2020). “NordicAktiva”. v1.3.0. NordicNeuroLab. PC.

[ref32] Papa A. Cristea M. McGuigan N. Tamariz M. (2021). Effects of verbal instruction vs. modelling on imitation and overimitation. Human. Soc. Sci. Commun. 8:239. doi: 10.1057/s41599-021-00925-4

[ref33] Perry D. G. Bussey K. (1979). The social learning theory of sex differences: imitation is alive and well. J. Pers. Soc. Psychol. 37, 1699–1712. doi: 10.1037/0022-3514.37.10.1699

[ref34] Putt S. S. Wijeakumar S. Franciscus R. G. Spencer J. P. (2017). The functional brain networks that underlie early stone age tool manufacture. Nat. Hum. Behav. 1:0102. doi: 10.1038/s41562-017-0102

[ref35] Putt S. S. Wijeakumar S. Spencer J. P. (2019). Prefrontal cortex activation supports the emergence of early stone age toolmaking skill. NeuroImage 199, 57–69. doi: 10.1016/j.neuroimage.2019.05.056, PMID: 31128246

[ref36] Raczkowski D. Kalat J. W. Nebes R. (1974). Reliability and validity of some handedness questionnaire items. Neuropsychologia 12, 43–47. doi: 10.1016/0028-3932(74)90025-6, PMID: 4821188

[ref37] Rueda M. R. (2018). Attention in the heart of intelligence. Trends Neurosci. Educ. 13, 26–33. doi: 10.1016/j.tine.2018.11.003

[ref38] Salagnon M. D’errico F. Mellet E. (2020). Neuroimaging and neuroarchaeology: a window on cognitive evolution. Intellectica-La revue de l’Association pour la Recherche sur les sciences de la Cognition (ARCo) 73, 67–91. doi: 10.3406/intel.2020.1965

[ref39] Sehasseh E. M. Fernandez P. Kuhn S. Stiner M. Mentzer S. Colarossi D. . (2021). Early middle stone age personal ornaments from Bizmoune Cave, Essaouira, Morocco. Sci. Adv. 7:eabi8620. doi: 10.1126/sciadv.abi8620, PMID: 34550742 PMC8457661

[ref40] Shutts K. Banaji M. R. Spelke E. S. (2010). Social categories guide young children’s preferences for novel objects. Dev. Sci. 13, 599–610. doi: 10.1111/j.1467-7687.2009.00913.x, PMID: 20590724 PMC2898520

[ref41] Soffer O. Adovasio J. M. (2010). “The roles of perishable technologies in upper Paleolithic lives” in The Magdalenian household: unraveling domesticity. eds. Zubrow E. B. W. Audouze F. Enloe J. G. (New York: SUNY Press).

[ref42] Stout D. (2021). The cognitive science of technology. Trends Cogn. Sci. 25, 964–977. doi: 10.1016/j.tics.2021.07.005, PMID: 34362661

[ref43] Stout D. Chaminade T. (2007). The evolutionary neuroscience of tool making. Neuropsychologia 45, 1091–1100. doi: 10.1016/j.neuropsychologia.2006.09.014, PMID: 17070875

[ref44] Stout D. Hecht E. E. (2017). Evolutionary neuroscience of cumulative culture. Proc. Natl. Acad. Sci. 114, 7861–7868. doi: 10.1073/pnas.1620738114, PMID: 28739892 PMC5544267

[ref45] Stout D. Hecht E. Khreisheh N. Bradley B. Chaminade T. (2015). Cognitive demands of lower Paleolithic toolmaking. PLoS One 10:e0121804. doi: 10.1371/journal.pone.0121804, PMID: 25875283 PMC4398452

[ref46] Stout D. Passingham R. Frith C. Apel J. Chaminade T. (2011). Technology, expertise and social cognition in human evolution. Eur. J. Neurosci. 33, 1328–1338. doi: 10.1111/j.1460-9568.2011.07619.x, PMID: 21375598

[ref47] Stout D. Toth N. Schick K. Chaminade T. (2008). Neural correlates of early stone age toolmaking: technology, language and cognition in human evolution. Philos. Trans. Roy. Soc. B Biol. Sci. 363, 1939–1949. doi: 10.1098/rstb.2008.0001, PMID: 18292067 PMC2606694

[ref48] Tennie C. Call J. Tomasello M. (2009). Ratcheting up the ratchet: on the evolution of cumulative culture. Philos. Trans. Roy. Soc. B Biol. Sci. 364, 2405–2415. doi: 10.1098/rstb.2009.0052, PMID: 19620111 PMC2865079

[ref49] Tomasello M. (1999). The cultural origins of human cognition. Cambridge, MA: Harvard University Press.

[ref50] Tomasello M. Kruger A. C. Ratner H. H. (1993). Cultural learning. Behav. Brain Sci. 16, 495–511. doi: 10.1017/S0140525X0003123X

[ref51] Uomini N. Lawson R. (2017). Effects of handedness and viewpoint on the imitation of origami-making. Symmetry 9:182. doi: 10.3390/sym9090182

[ref52] Vanhaeren M. d'Errico F. Stringer C. James S. L. Todd J. A. Mienis H. K. (2006). Middle Paleolithic shell beads in Israel and Algeria. Science 312, 1785–1788. doi: 10.1126/science.1128139, PMID: 16794076

[ref53] Vanhaeren M. d'Errico F. Van Niekerk K. L. Henshilwood C. S. Erasmus R. M. (2013). Thinking strings: additional evidence for personal ornament use in the middle stone age at Blombos Cave, South Africa. J. Hum. Evol. 64, 500–517. doi: 10.1016/j.jhevol.2013.02.001 23498114

[ref54] Vogt S. Buccino G. Wohlschläger A. M. Canessa N. Shah N. J. Zilles K. . (2007). Prefrontal involvement in imitation learning of hand actions: effects of practice and expertise. NeuroImage 37, 1371–1383. doi: 10.1016/j.neuroimage.2007.07.005, PMID: 17698372

[ref55] Wasielewski H. (2014). Imitation is necessary for cumulative cultural evolution in an unfamiliar, opaque task. Hum. Nat. 25, 161–179. doi: 10.1007/s12110-014-9192-5, PMID: 24519404

[ref56] Watanabe R. Higuchi T. Kikuchi Y. (2013). Imitation behavior is sensitive to visual perspective of the model: an fMRI study. Exp. Brain Res. 228, 161–171. doi: 10.1007/s00221-013-3548-7, PMID: 23660743

[ref8002] Wellcome Centre for Human Neuroimaging. (2020). “SPM12”. v7771. Wellcome Centre for Human Neuroimaging. PC.

[ref57] Xu J. Wang J. Fan L. Li H. Zhang W. Hu Q. . (2015). Tractography-based parcellation of the human middle temporal gyrus. Sci. Rep. 5:18883. doi: 10.1038/srep18883, PMID: 26689815 PMC4686935

[ref58] Zwirner E. Thornton A. (2015). Cognitive requirements of cumulative culture: teaching is useful but not essential. Sci. Rep. 5, 1–8. doi: 10.1038/srep16781, PMID: 26606853 PMC4660383

