# Supplementary material for: The neural underpinnings of repeated skill transfer in human cultural evolution
Source: Front Psychol. 2025 May 7;16:1545120. doi: 10.3389/fpsyg.2025.1545120 (PMC12092357; doi:10.3389/fpsyg.2025.1545120)
Supplement: Supplementary file 1 [file Supplementary_file_1.docx]

Supplementary Material


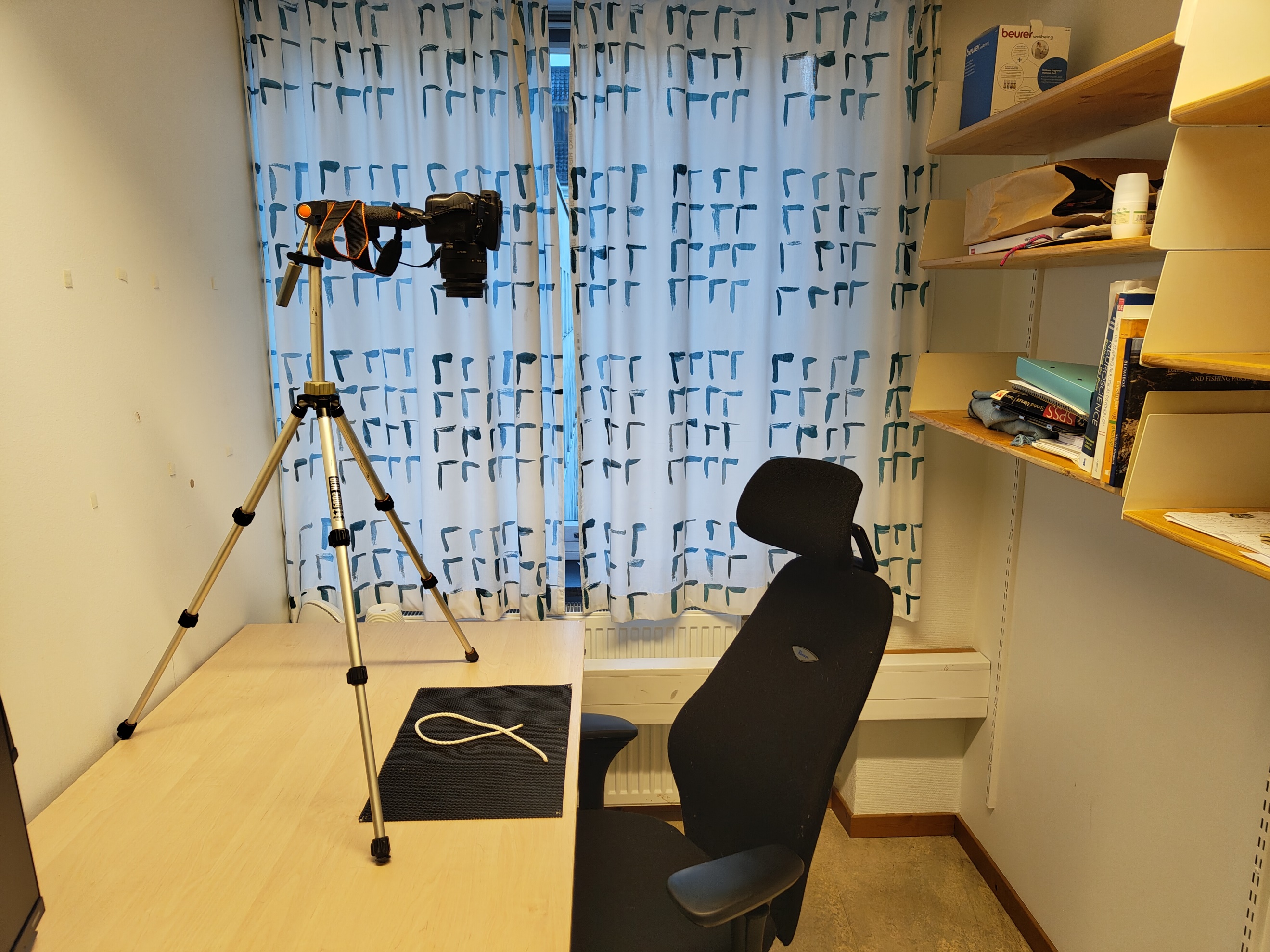


Supplementary figure 1: Camera setup for filming the video demonstrations.


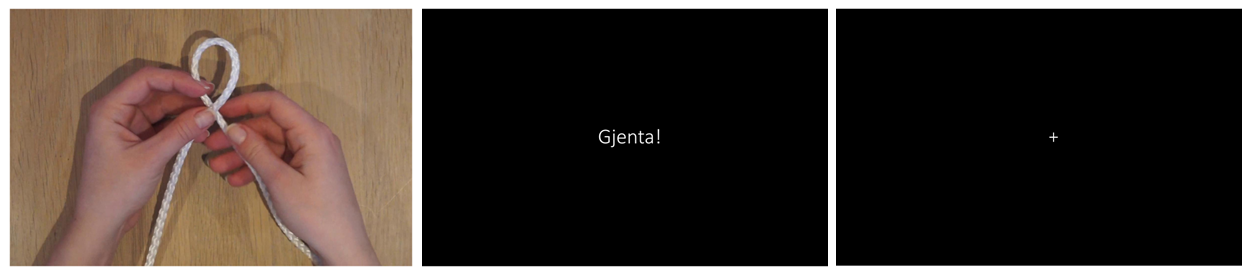
 Supplementary figure 2: Examples of the types of images participants saw on the screen during the experimental procedure.

Supplementary table 2: Average framewise displacement for all participants separated by knot

|  | mean FD (SD) | | | | | |
| --- | --- | --- | --- | --- | --- | --- |
| ID | Control | Figure eight knot | Noose knot | Sheet bend | Slip knot | Square knot |
| C1G1 | 0.187 ± 0.09 | 0.172 ± 0.095 | 0.182 ± 0.092 | 0.207 ± 0.121 | 0.189 ± 0.102 | 0.195 ± 0.095 |
| C1G2 | 0.125 ± 0.067 | 0.123 ± 0.065 | 0.227 ± 0.186 | 0.142 ± 0.106 | 0.13 ± 0.077 | 0.171 ± 0.113 |
| C1G3 | 0.145 ± 0.124 | 0.276 ± 0.296 | 0.189 ± 0.15 | 0.282 ± 0.31 | 0.216 ± 0.19 | 0.194 ± 0.15 |
| C1G4 | 0.204 ± 0.225 | 0.306 ± 0.44 | 0.218 ± 0.241 | 0.229 ± 0.242 | 0.232 ± 0.303 | 0.304 ± 0.345 |
| C1G5 | 0.111 ± 0.045 | 0.105 ± 0.038 | 0.142 ± 0.073 | 0.144 ± 0.075 | 0.114 ± 0.056 | 0.133 ± 0.058 |
| C1G6 | 0.104 ± 0.06 | 0.135 ± 0.096 | 0.11 ± 0.07 | 0.113 ± 0.083 | 0.132 ± 0.095 | 0.16 ± 0.134 |
| C1G7 | 0.14 ± 0.063 | 0.14 ± 0.079 | 0.163 ± 0.118 | 0.157 ± 0.086 | 0.163 ± 0.112 | 0.162 ± 0.16 |
| C1G8 | 0.141 ± 0.08 | 0.134 ± 0.082 | 0.179 ± 0.14 | 0.165 ± 0.1 | 0.182 ± 0.138 | 0.144 ± 0.082 |
| C2G1 | 0.131 ± 0.096 | 0.135 ± 0.095 | 0.168 ± 0.146 | 0.143 ± 0.098 | 0.148 ± 0.108 | 0.118 ± 0.084 |
| C2G2 | 0.223 ± 0.117 | 0.193 ± 0.115 | 0.22 ± 0.129 | 0.208 ± 0.13 | 0.215 ± 0.129 | 0.235 ± 0.159 |
| C2G3 | 0.193 ± 0.291 | 0.116 ± 0.05 | 0.116 ± 0.064 | 0.121 ± 0.063 | 0.124 ± 0.068 | 0.112 ± 0.057 |
| C2G4 | 0.155 ± 0.144 | 0.15 ± 0.078 | 0.116 ± 0.089 | 0.129 ± 0.075 | 0.181 ± 0.115 | 0.163 ± 0.105 |
| C2G5 | 0.132 ± 0.058 | 0.136 ± 0.06 | 0.116 ± 0.073 | 0.154 ± 0.069 | 0.148 ± 0.073 | 0.135 ± 0.068 |
| C2G6 | 0.088 ± 0.055 | 0.101 ± 0.067 | 0.116 ± 0.075 | 0.1 ± 0.057 | 0.109 ± 0.081 | 0.104 ± 0.06 |
| C2G7 | 0.126 ± 0.064 | 0.176 ± 0.135 | 0.116 ± 0.14 | 0.185 ± 0.137 | 0.183 ± 0.185 | 0.185 ± 0.15 |

Average framewise displacement (FD) calculated using the formula of Power and colleagues (2012).

Reference: Power, J. D., Barnes, K. A., Snyder, A. Z., Schlaggar, B. L. & Petersen, S. E. Spurious but systematic correlations in functional connectivity MRI networks arise from subject motion. Neuroimage 59, 2142-2154 (2012).

Supplementary table 1: Knot evaluation scores for how well the participants managed to reproduce each of the knots they were shown. Scores of 3 or lower are counted as wrong, and scores of 4 and 5 are counted as correct.

|  |  | Chain 1 | | | | | | | | |  | | Chain 2 | | | | | | | | |
| --- | --- | --- | --- | --- | --- | --- | --- | --- | --- | --- | --- | --- | --- | --- | --- | --- | --- | --- | --- | --- | --- |
| Generation |  | 1 | 2 | 3 | 4 | 5 | 6 | 7 | 8 |  | | 1 | | 2 | 3 | 4 | 5 | 6 | 7 | 8 |  |
| Fig8 |  | 5 | 5 | 5 | 3 | 5 | 5 | 5 | 5 |  | | 5 | | 5 | 5 | 5 | 5 | 5 | 5 | - |  |
| Noose |  | 5 | 4 | 4 | 4 | 5 | 5 | 5 | 5 |  | | 5 | | 3 | 5 | 4 | 5 | 5 | 5 | - |  |
| Sheet |  | 5 | 4 | 5 | 3 | 3 | 3 | 3 | 3 |  | | 5 | | 3 | 3 | 3 | 5 | 5 | 5 | - |  |
| Slip |  | 4 | 5 | 5 | 5 | 5 | 2 | 5 | 5 |  | | 5 | | 4 | 5 | 5 | 3 | 3 | 3 | - |  |
| Square |  | 5 | 5 | 4 | 1 | 5 | 2 | 5 | 5 |  | | 5 | | 3 | 5 | 5 | 5 | 5 | 5 | - |  |
